# Supplementary material for: A divisive model of evidence accumulation explains uneven weighting of evidence over time
Source: Nat Commun. 2020 May 1;11:2160. doi: 10.1038/s41467-020-15630-0 (PMC7195479; doi:10.1038/s41467-020-15630-0)
Supplement: Supplementary file 1 — Supplementary Information [file 41467_2020_15630_MOESM1_ESM.pdf]

Supplementary Information

A divisive model of evidence accumulation explains uneven weighting of evidence over time

Keung et al.

Supplementary Note 1

Errors contributed by uneven integration kernel

To understand how much error an uneven integration kernel introduces, we estimated error rates using simulations that keep the uneven integration kernel as the only source of error.

We first used logistic regression to estimate the regression weights of each click for each participant, as described in Main Text equation (8), with the equation replicated here:

$$\text{logit}(p_{\text{left}} \text{ at trial } t) = \sum_{i=1}^{20} \beta_i^{\text{click}} \Delta C_i + \beta^{\text{bias}} \tag{1}$$

β_i^{click} is the weight of the ith click on choice, and β^{bias} is the weight of an overall side bias (i.e. the weight of always choosing left).

We then simulated participants' choices by reversing the logistic regression — for each participant, using the estimated betas, we computed p_{left}, the probability of that participant choosing left on a given trial, using the same equation (Supplementary Equation (1)). We then reproduced the participant's choices by randomly drawing from a binomial distribution with the computed p_{left} for each trial.

Then we compared the error rate of the simulated choices to participants' actual choices. We showed in the rightmost two data points in Supplementary Figure 1 that we reproduced the same total error rate in simulations using the original estimated regression weights (20.6%) as the total error rate in human participants' data (20.2%).

We then removed the overall side bias by setting β^{side} to be zero. We also removed overall noise by making the choices deterministic. That is, instead of randomly drawing from a binomial distribution using p_{left}, we asked whether $\text{logit}(p_{\text{left}})$ at a given trial is larger or smaller than zero: if $\text{logit}(p_{\text{left}})$ is larger than zero, choose left, and if $\text{logit}(p_{\text{left}})$ is smaller than zero, choose right. If $\text{logit}(p_{\text{left}})$ is equal to zero, then flip a coin with 50% probability. By removing these other sources of errors, we asked how much errors the uneven integration kernel shape contributes to. We showed that an uneven integration kernel shape contributed to 5.5% error rate (Supplementary Figure 1), which accounted

for 27.2% of the total error rate. We also showed that by removing the uneven integration kernel, simulations showed zero error rates.

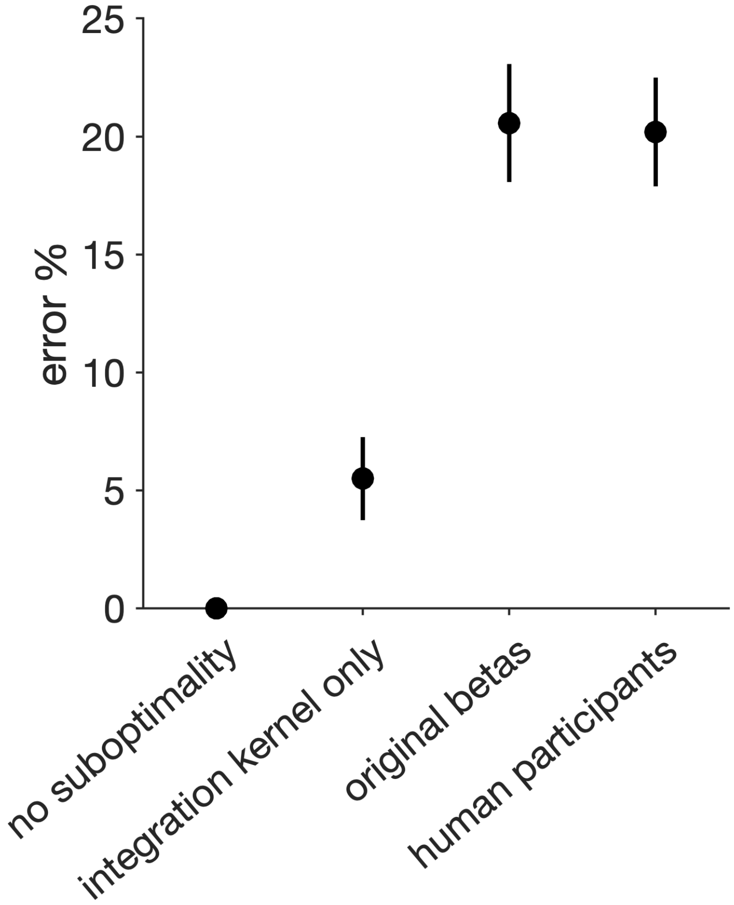

Supplementary Figure 1: Error rates from simulated choices compared to human participants. From left to right: error rates of simulated choices with 1) no suboptimalities, 2) only uneven integration kernel, and 3) original betas, and error rate of human participants. Error bars indicate s.e.m. across participants.

Supplementary Note 2

Categorizing integration kernels into shapes

To categorize the kernel for each participant into one of the four shapes, we fit polynomial functions with different degrees to participants' choices, and selected the best fitting model with model comparison using the Akaike Information Criterion (AIC) to account for the different number of free parameters. In particular, we assume that the probability of choosing left at trial t is (the logit of) the weighted sum of clicks, where the weights are from a polynomial function, as shown in the following equation:

$$\text{logit}(p_{\text{left}} \text{ at trial } t) = \sum_{i=1}^{20} \beta_i^{\text{poly}} \Delta C_i \text{ , where } \beta_i^{\text{poly}} = \sum_{n=0}^{N} \alpha_n i^n \tag{2}$$

We fitted three different polynomial functions by changing N from 0 to 2: constant, linear, and quadratic. We then selected the best fitting function for each participant by comparing the fits from different polynomials with AIC. We categorized each participant's integration kernel into one of the four shapes using the following criteria: (1) flat: kernel was best fit with the constant function; (2) primacy: kernel was best fit with linear function with a negative slope (α_1), or with quadratic function with a minimum ($\alpha_2 > 0$) and the minimum is located later than the 10th click, or with quadratic function with a maximum ($\alpha_2 < 0$) and the maximum is located earlier than the 2nd click; (3) recency: kernel was best fit with linear function with a positive slope, or with quadratic function with a minimum ($\alpha_2 > 0$) and the minimum is located earlier than the 10th click, or with quadratic function with a maximum ($\alpha_2 < 0$) and the maximum is located later than the 18th click; (4) bump: kernel that did not meet the previous three criteria (i.e. kernel was best fit with quadratic function and was neither primacy nor recency). Individual integration kernels and their categorizations are plotted in Supplementary Figure 2.

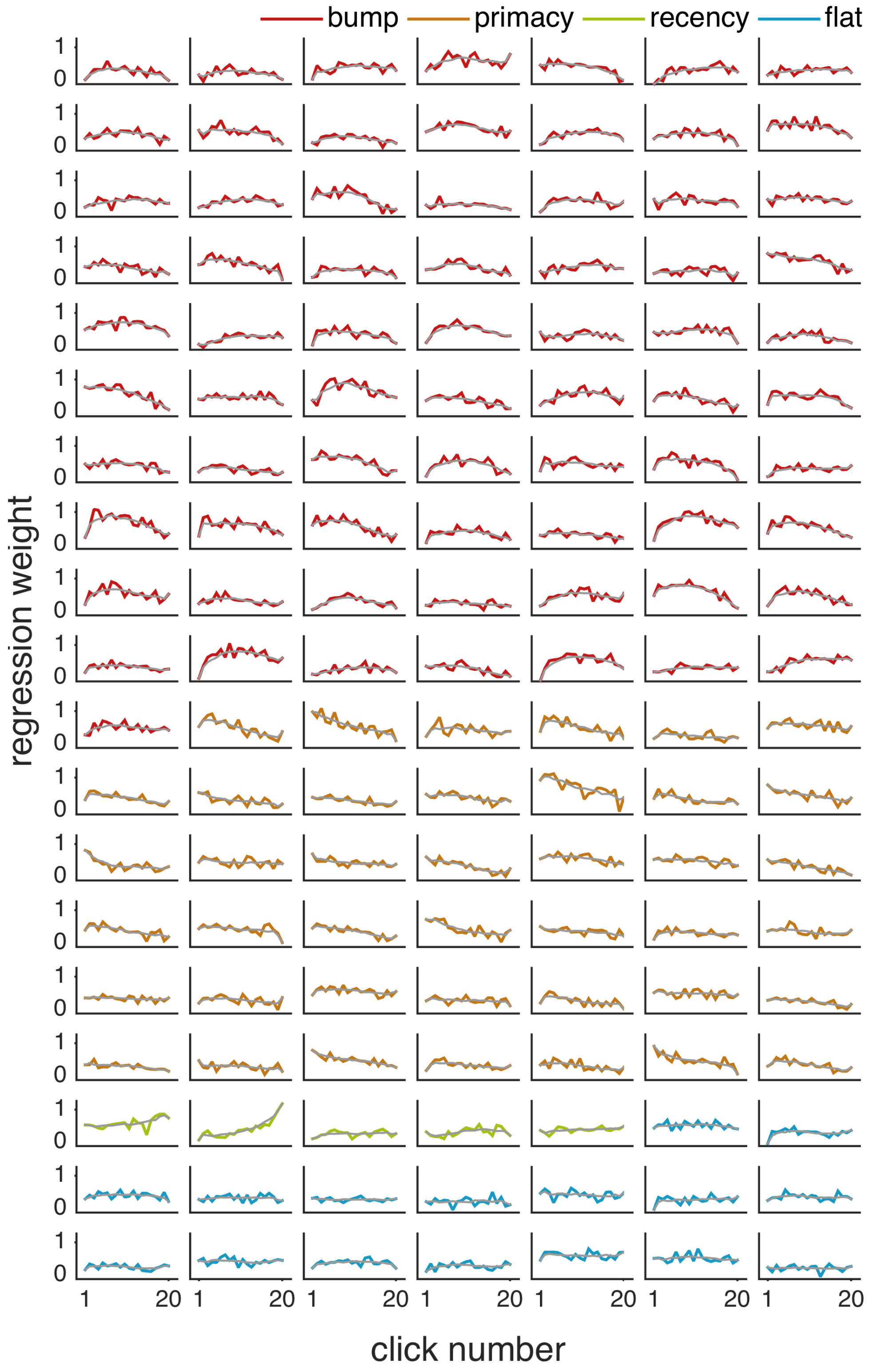

Supplementary Figure 2: Individual integration kernel plots. Colored lines are regression weights of clicks from equation (8). Plots are sorted and color coded by kernel shape. Light grey line shows smoothed integration kernel.

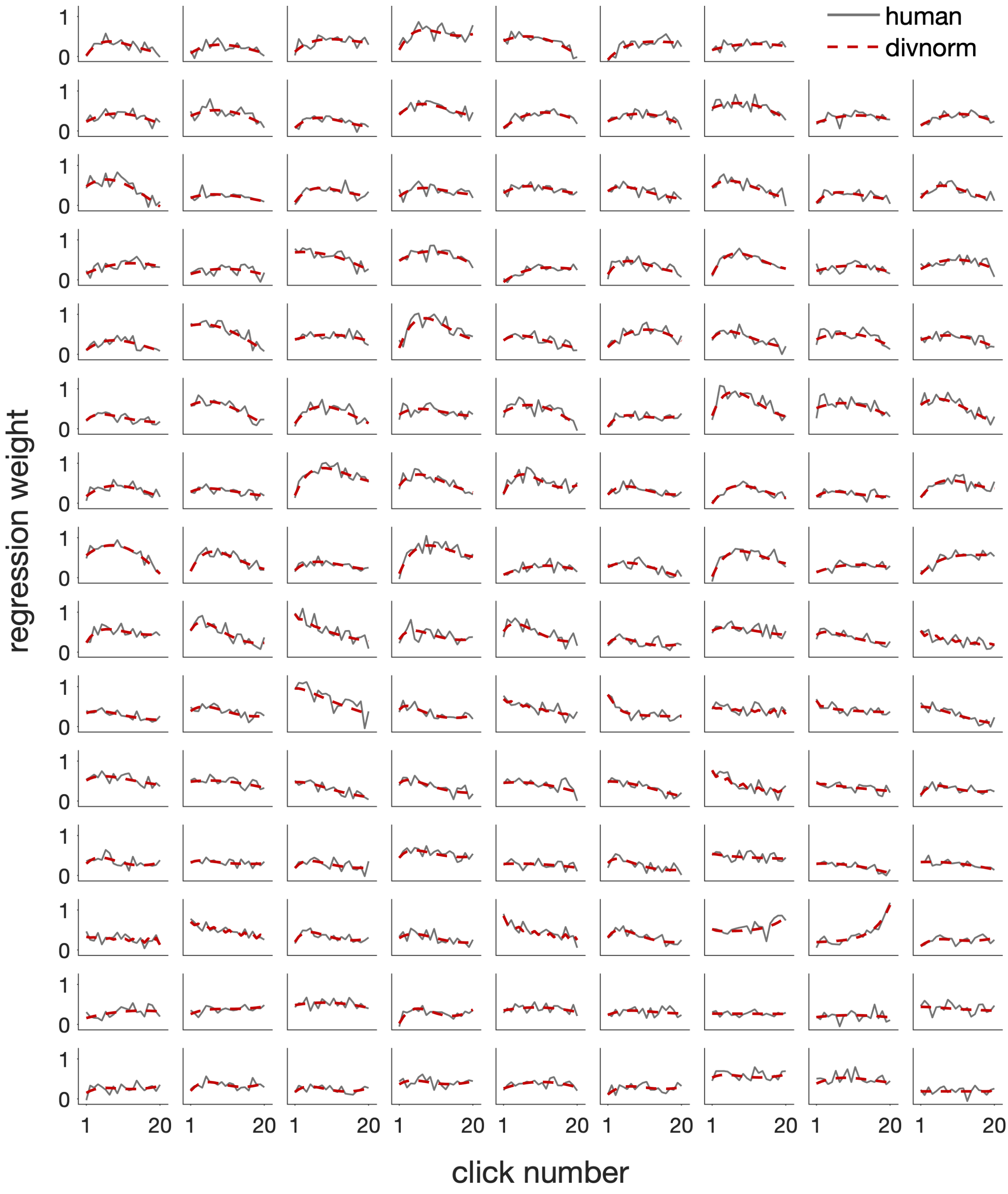

Supplementary Figure 3: Individual integration kernels generated by divisive normalization compared to human integration kernels.

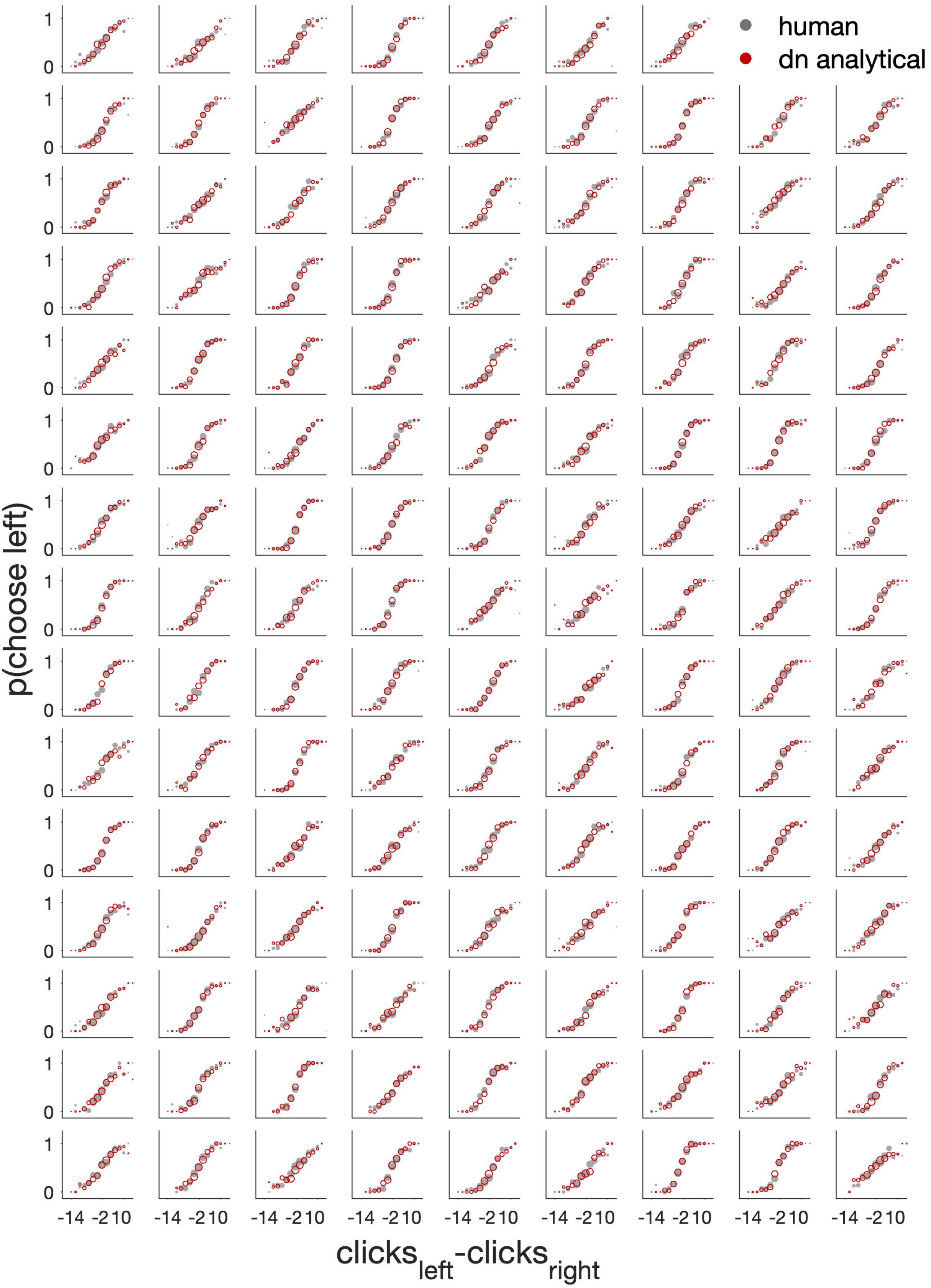

Supplementary Figure 4: Individual psychometric curves generated by divisive normalization compared to human psychometric curves.

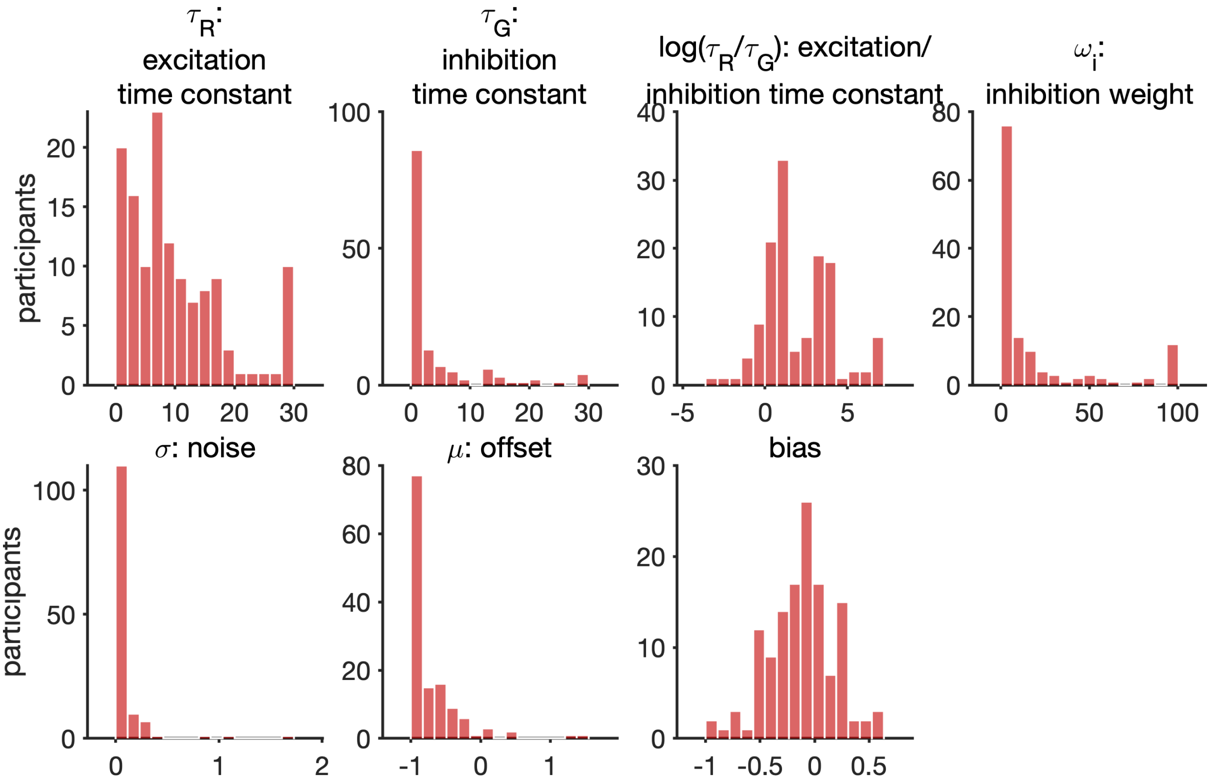

Supplementary Figure 5: Histogram of fitted divisive normalization parameters.

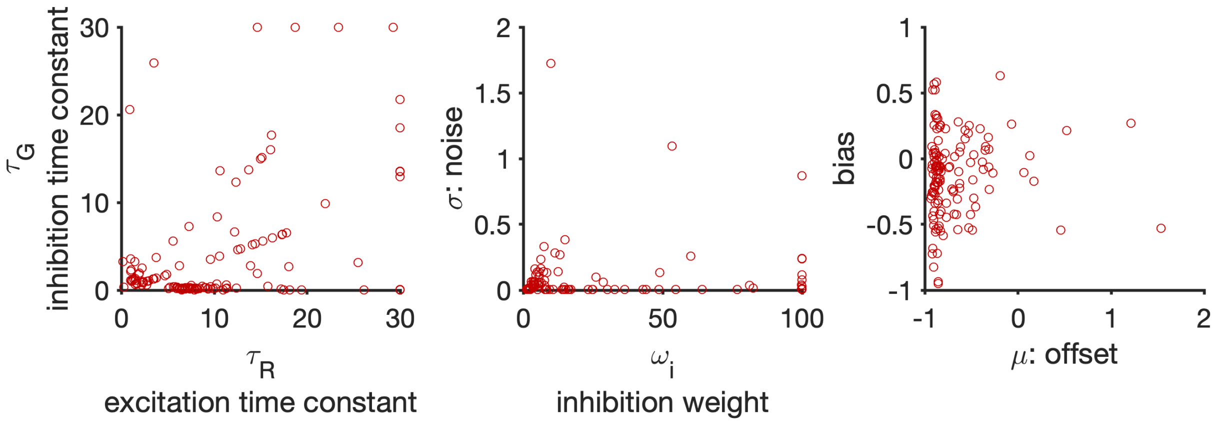

Supplementary Figure 6: Scatter plots of fitted divisive normalization parameters, plotted in pairs of parameters.

Supplementary Note 3

LCA does not fit bump kernel

We first tested how LCA fits to the behavioural data. We fitted LCA to participants' choices using a maximum likelihood approach. An analytical solution of the probability of choosing a certain side exists for LCA under fixed decision time protocol [1]. Specifically, assuming the probability distribution of the initial accumulator state is Gaussian (with initial mean μ_0 and initial variance v_0), the probability distribution of the accumulator state at the end of the stimulus train is also Gaussian, and the mean and variance can be computed analytically. Thus, the probability of choosing one side is the cumulative normal distribution.

We used maximum likelihood approach to fit these five parameters in LCA: initial mean μ_0, initial variance v_0, memory drift λ, noise σ, and an overall bias. We generated choices from the model using best fitting parameters. Integration kernels from LCA can be reconstructed by regressing the stimulus onto model generated choices using logistic regression (equation (8)). Confirming our intuition, we show that LCA can produce a primacy, recency, and flat effect, but cannot fit to the bump kernel (Supplementary Figure 7a).

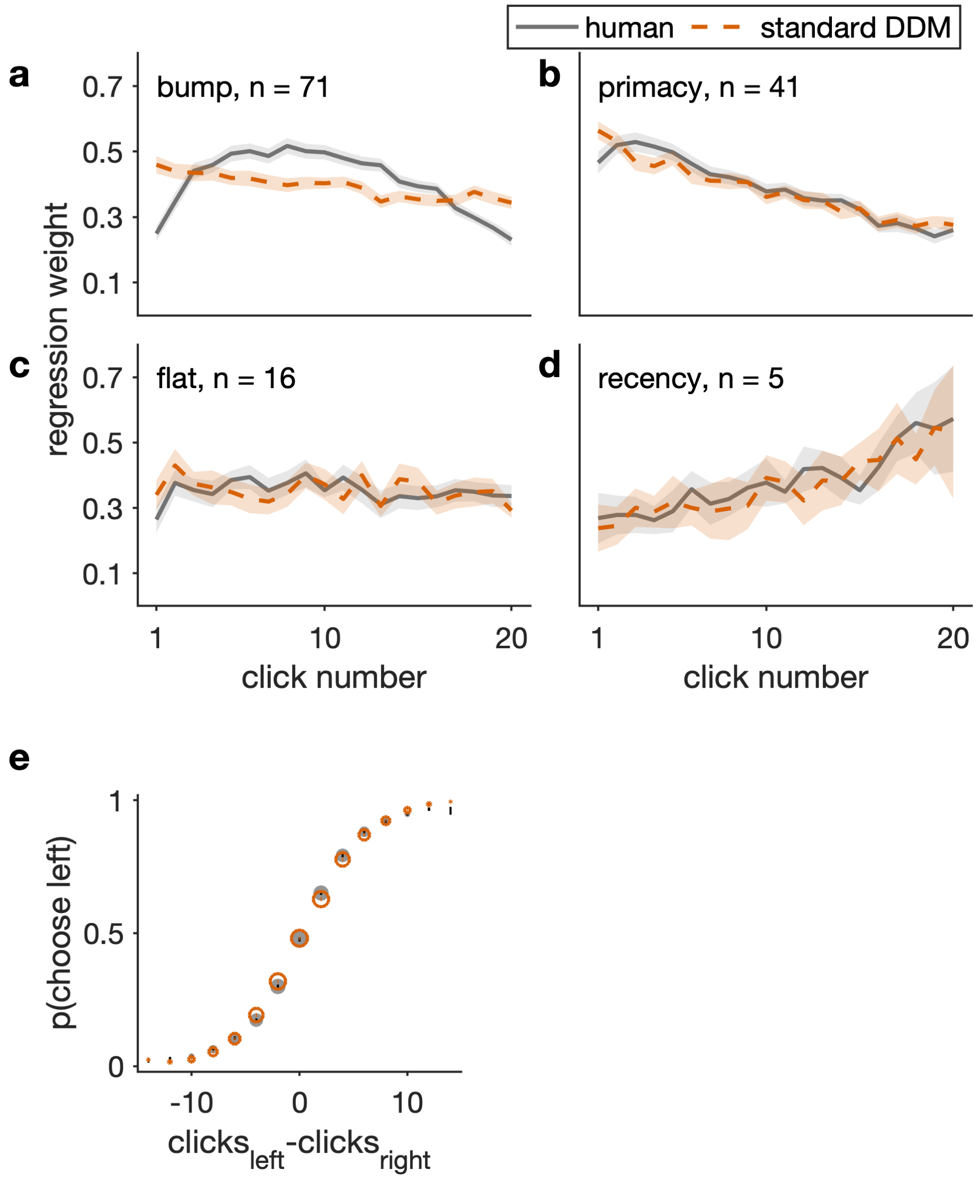

Supplementary Figure 7: LCA cannot account for bump shaped kernel. (a-d) Integration kernels generated by LCA, compared to human integration kernels. Plots are grouped into groups of four different integration kernel shapes. Grey line indicates human integration kernels. Orange line indicates model generated kernels. All shaded areas indicate s.e.m. across participants. (e) Psychometric curves generated by LCA compared to human psychometric curve. Grey circle indicates human psychometric curve. Orange circle indicates model generated psychometric curve. Error bars indicate s.e.m. across participants.

Supplementary Note 4

6 parameter Brunton et al. DDM fits participants' behaviour

We then fitted the Brunton et al. DDM [2] to the behavioural data, following code by [3]. The key differences between the Brunton et al. DDM and standard LCA model is the addition of two processes. The first is a 'sticky' bound — that is, a decision is made either at the end of the stimulus train, or at the time when the accumulator hits the bound, depending on which event happens earlier. The second is a sensory adaptation process which controls the actual impact of a click (without adaptation the impact of every click will always be 1). The process is controlled by two parameters: 1) the direction of adaptation — either no adaptation, depression (the impact of each click is smaller than 1, or facilitation (the impact is larger than 1), and 2) a time constant that determines how quickly the adapted impact recovers to 1.

To fit the Brunton el al. model, the probability distribution of accumulator state evolves over time following a similar logic to the standard DDM. To model the bound in the probability distribution space, a 'sticky' bound is added to DDM such that when the probability mass hits the bound, it sticks to the bound. The evolution of the probability distribution for each trial has to be computed numerically, until the end of the stimulus. Similar to the case of standard DDM, the probability of choosing one side is the cumulative distribution of the probability distribution at the end of the stimulus train. This cumulative distribution is also computed numerically. We fitted the model to participants' choices using code provided in [3]. We show that Brunton DDM fits to all four integration kernel shapes (Supplementary Figure 8).

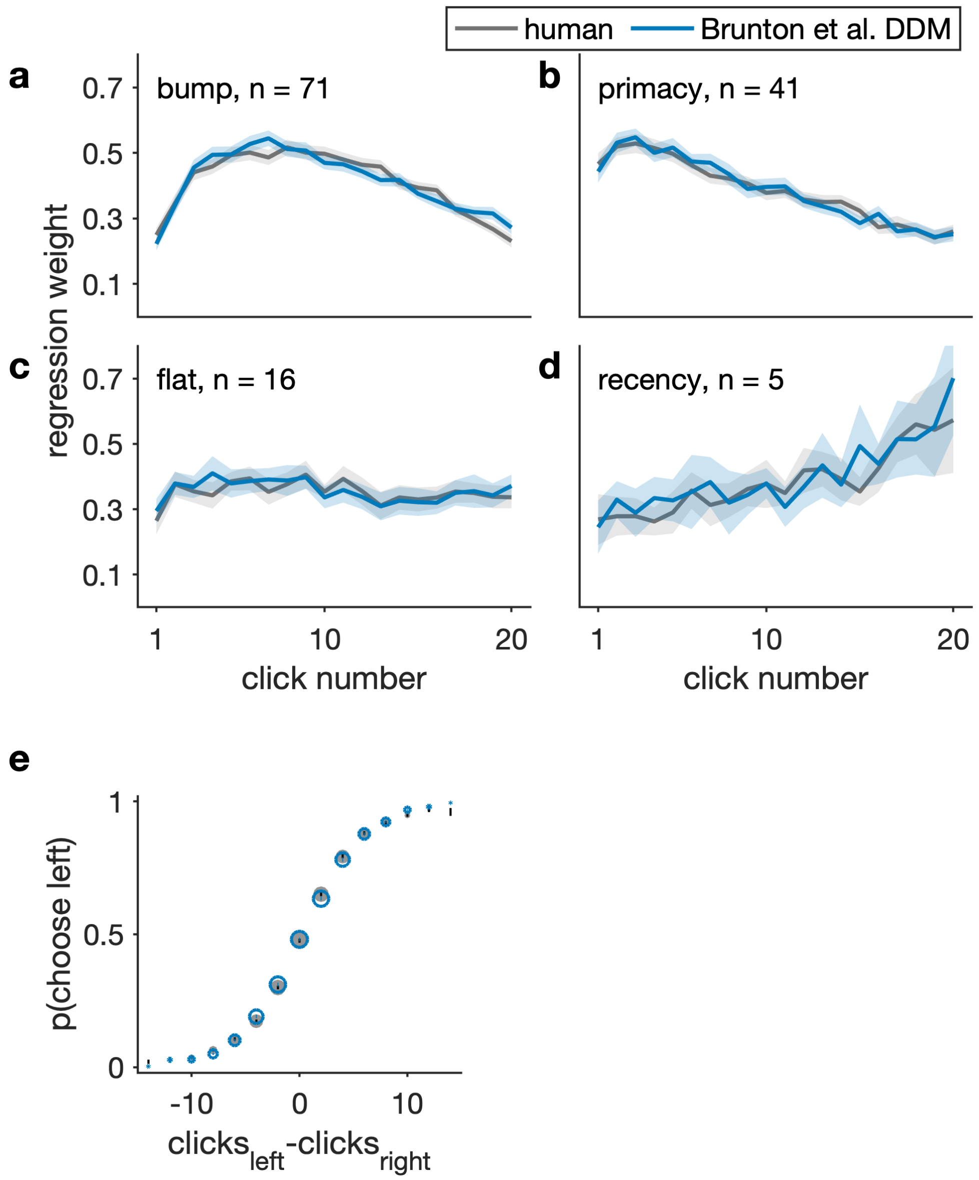

Supplementary Figure 8: Brunton et al. 6 param DDM accounts for human integration kernels. (a-d) Integration kernels generated by Brunton et al. model, compared to human integration kernels. Plots are grouped into groups of four different integration kernel shapes. Grey line indicates human integration kernels. Blue line indicates model generated kernels. All shaded areas indicate s.e.m. across participants. (e) Psychometric curves generated by Brunton et al. model compared to human psychometric curve. Grey circle indicates human psychometric curve. Blue circle indicates model generated psychometric curve. Error bars indicate s.e.m. across participants.

Supplementary Note 5

9 parameter Brunton et al. DDM fits behaviour as well as 6 parameter model does.

The original model reported in Brunton et al. [2] has three additional parameters: 1) an additional noise parameter σ_s that characterizes the noise added at each incoming stimulus (i.e. click), 2) a noise parameter σ_i that characterizes the amount of noise in the initial state of the accumulator, and 3) a lapse rate that characterizes the probability of a random response being made. We fitted the Brunton et al. DDM with nine parameters to our behavioural data and show that the DDM fit to the data as well as the DDM with six parameters (Supplementary Figure 9).

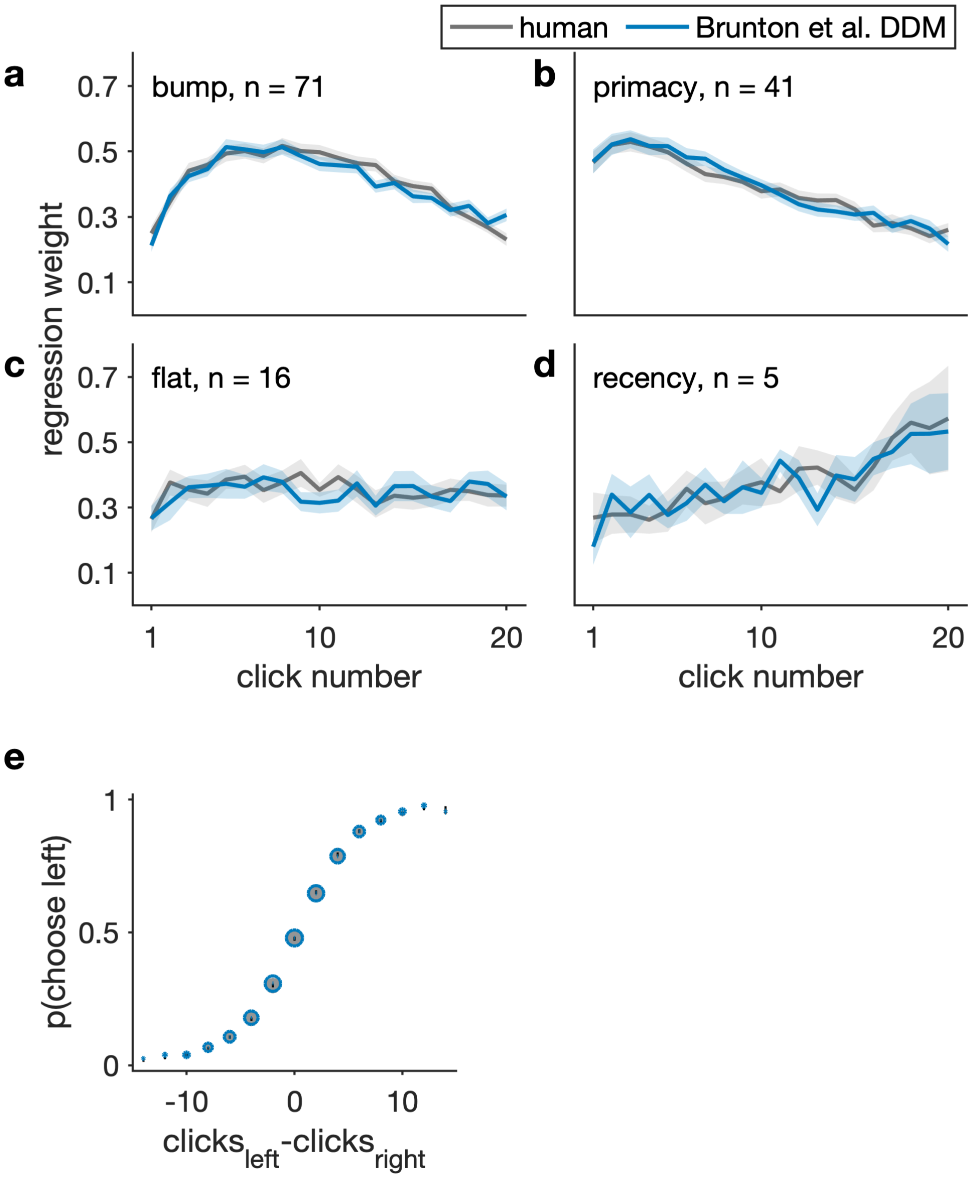

Supplementary Figure 9: 9 parameter Brunton et al. DDM fits behaviour as well as 6 param does. (a-d) Integration kernels generated by Brunton et al. 9 parameter model, compared to human integration kernels. Plots are grouped into groups of four different integration kernel shapes. Grey line indicates human integration kernels. Blue line indicates model generated kernels. All shaded areas indicate s.e.m. across participants. (e) Psychometric curves generated by Brunton et al. 9 parameter model compared to human psychometric curve. Grey circle indicates human psychometric curve. Blue circle indicates model generated psychometric curve. Error bars indicate s.e.m. across participants.

Supplementary Note 6

LCA with bound does not fit bump kernel

We also fitted a LCA adding only a bound. This is essentially the aforementioned 6 parameter Brunton et al. DDM minus the sensory adaptation component. A total of four parameters were fitted: memory drift λ, noise σ, bias, and bound. We showed that the LCA with just a bound does not account for the bump shaped kernel. (Supplementary Figure 10).

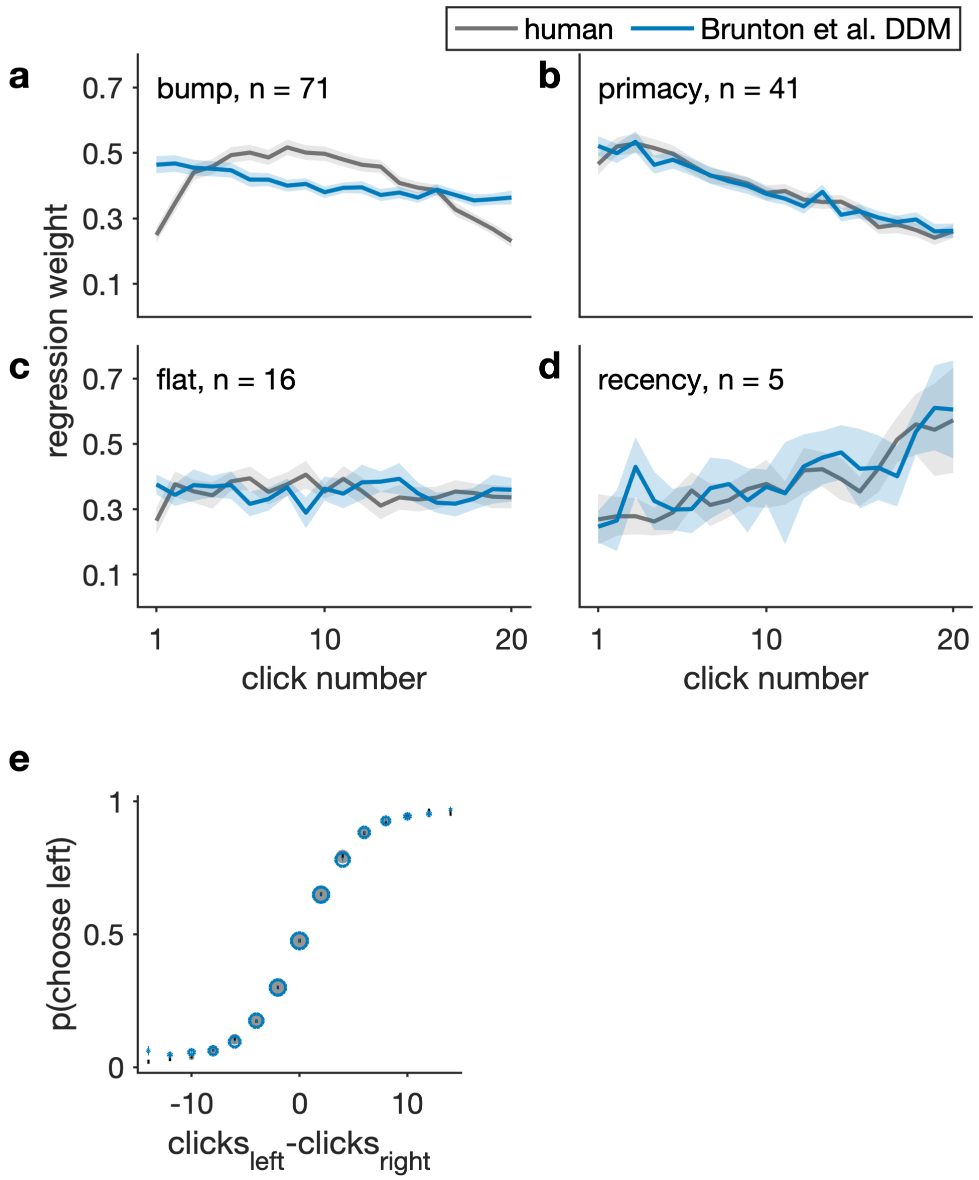

Supplementary Figure 10: LCA with just a bound cannot account for bump shaped kernel. (a-d) Integration kernels generated by LCA, compared to human integration kernels. Plots are grouped into groups of four different integration kernel shapes. Grey line indicates human integration kernels. Blue line indicates model generated kernels. All shaded areas indicate s.e.m. across participants. (e) Psychometric curves generated by LCA with bound compared to human psychometric curve. Grey circle indicates human psychometric curve. Blue circle indicates model generated psychometric curve. Error bars indicate s.e.m. across participants.

Model	Log likelihood	AIC	BIC
Divisive normalization (6 param)	-357.8	727.5	755.2
LCA [1]	-364.2	738.5	761.6
Brunton et al. DDM (6 param) [2]	-358.5	729.5	756.8
Brunton et al. DDM (9 param) [2]	-357.7	733.1	774.7
LCA with bound [2]	-362.2	738.4	770.8

Supplementary Table 1: Formal model comparison. Log likelihood, AIC, and BIC scores of all models tested.

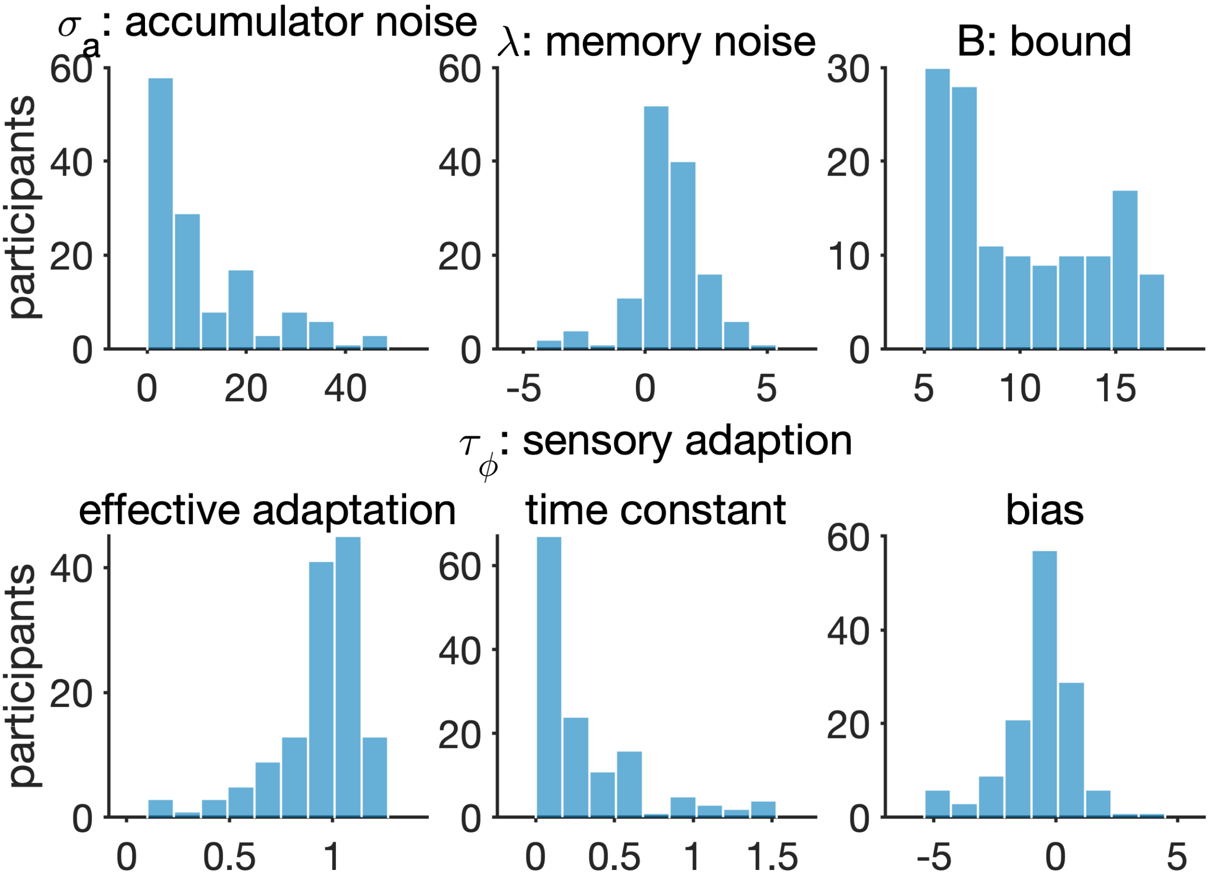

Supplementary Figure 11: Histogram of fitted parameters of 6 param Brunton DDM.

Supplementary Note 7

Attractor space of divisive normalization

We show that there is one attractor in the A-G space where A is the total network activity:

$$A = R_{\text{left}} + R_{\text{right}} \tag{3}$$

Since with our experimental design, there is always a click input at each time point (so either $C_{\text{left}} = 1$ or $C_{\text{right}} = 1$), the sum of inputs into the network is constant at 1 over time. Thus, the set of differential equations for the A-G space are:

$$\tau_R \frac{dA}{dt} = -A + \frac{1}{1+G} \tag{4}$$

$$\tau_G \frac{dG}{dt} = -G + \omega_{\text{I}} \sum_{i=1}^{N} R_i = -G + \omega_{\text{I}} A \tag{5}$$

Analytically, a full proof for the existence of a unique equilibrium point that is asymptotically stable for general families of this set of differential equations was provided by Louie, et al. [4]. Numerically, we produce plots of vector field and model trajectory to demonstrate the stable equilibrium point for divisive normalization in the A-G space (Supplementary Figure 12).

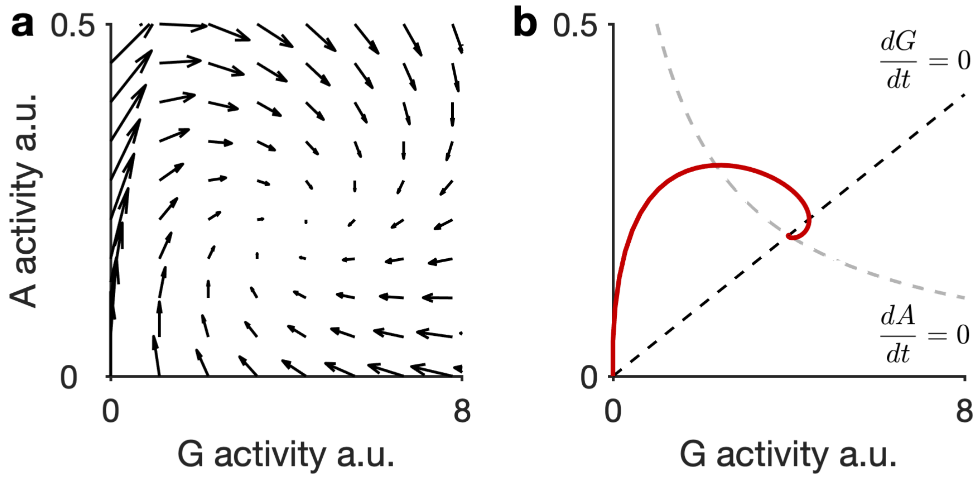

Supplementary Figure 12: Characteristic model dynamics simulated with $\tau_R = 1$, $\tau_G = 1$, and $\omega_\mathrm{I} = 20$. (a) Example model vector field. Arrows indicate instantaneous change in the activities of the (A, G) pair for certain values of A and G. The vector field shows a stable equilibrium point in the A-G space. (b) Example network activity trajectory corresponding to the vector field in (a), indicated by red solid line. Grey and black dashed lines indicate the nullclines of the two differential equations for A and G respectively. The nullclines show values at which either A or G activity does not experience a change in activity (regardless of change in the activity in the other component). The intersection of the two nullclines is where neither the activity of A nor G changes, again indicating the stable equilibrium point of the network.

Supplementary References

[1] Feng, S., Holmes, P., Rorie, A. & Newsome, W. T. Can monkeys choose optimally when faced with noisy stimuli and unequal rewards? *PLoS computational biology* **5**, e1000284 (2009).

[2] Brunton, B. W., Botvinick, M. M. & Brody, C. D. Rats and humans can optimally accumulate evidence for decision-making. *Science* **340**, 95–98 (2013).

[3] Yartsev, M. M., Hanks, T. D., Yoon, A. M. & Brody, C. D. Causal contribution and dynamical encoding in the striatum during evidence accumulation. *Elife* **7**, e34929 (2018).

[4] Louie, K., LoFaro, T., Webb, R. & Glimcher, P. W. Dynamic divisive normalization predicts time-varying value coding in decision-related circuits. *Journal of Neuroscience* **34**, 16046–16057 (2014).
